# Exercise augmentation compared to usual care for Post Traumatic Stress Disorder: A Randomised Controlled Trial (The REAP study: Randomised Exercise Augmentation for PTSD)

**DOI:** 10.1186/1471-244X-11-115

**Published:** 2011-07-22

**Authors:** Simon Rosenbaum, Dang Nguyen, Tom Lenehan, Anne Tiedemann, Hidde P van der Ploeg, Catherine Sherrington

**Affiliations:** 1 St John of God Healthcare, Richmond Hospital 177 Grose Vale Rd North Richmond, NSW 2754, Australia; 2 Musculoskeletal Division, The George Institute for Global Health, The University of Sydney, PO Box M201, Missenden Rd, Sydney, NSW 2050, Australia; 3 Cluster for Physical Activity and Health, Sydney School of Public Health, Faculty of Medicine, Level 2 Medical Foundation Building (K25), The University of Sydney, NSW 2006, Australia

## Abstract

**Background:**

The physical wellbeing of people with mental health conditions can often be overlooked in order to treat the primary mental health condition as a priority. Exercise however, can potentially improve both the primary psychiatric condition as well as physical measures that indicate risk of other conditions such as diabetes mellitus and cardiovascular disease. Evidence supports the role of exercise as an important component of treatment for depression and anxiety, yet no randomised controlled trials (RCT's) have been conducted to evaluate the use of exercise in the treatment of people with post traumatic stress disorder (PTSD).

This RCT will investigate the effects of structured, progressive exercise on PTSD symptoms, functional ability, body composition, physical activity levels, sleep patterns and medication usage.

**Methods and design:**

Eighty participants with a Diagnostic and Statistical Manual of Mental Disorders (DSM-IV) diagnosis of PTSD will be recruited. Participants will have no contraindications to exercise and will be cognitively able to provide consent to participate in the study.

The primary outcome measures will be PTSD symptoms, measured through the PTSD Checklist Civilian (PCL-C) scale. Secondary outcome measures will assess depression and anxiety, mobility and strength, body composition, physical activity levels, sleep patterns and medication usage. All outcomes will be assessed by a health or exercise professional masked to group allocation at baseline and 12 weeks after randomisation.

The intervention will be a 12 week individualised program, primarily involving resistance exercises with the use of exercise bands. A walking component will also be incorporated. Participants will complete one supervised session per week, and will be asked to perform at least two other non-supervised exercise sessions per week. Both intervention and control groups will receive all usual non-exercise interventions including psychotherapy, pharmaceutical interventions and group therapy.

**Discussion:**

This study will determine the effect of an individualised and progressive exercise intervention on PTSD symptoms, depression and anxiety, mobility and strength, body composition, physical activity levels, sleep patterns and medication usage among people with a DSM-IV diagnosis of PTSD.

**Trial Registration:**

ACTRN12610000579099

## Background

Mental health consumers typically have poorer health outcomes than people of a comparable age without mental health issues and are more likely to have metabolic conditions such as diabetes, hypertension and hypercholesterolemia [1]. Regular exercise has been shown to positively impact upon factors contributing to the metabolic syndrome as well as improving depressive and anxiety related symptoms [2-4]. Despite these findings, and the potential 'double impact' that regular exercise may have on conditions such as PTSD, mental health consumers are less likely to embark on and adhere to a regular exercise program [5].

In order to maintain health and reduce the risk of chronic disease, the American College of Sports Medicine (ACSM) recommends adults perform moderately intense cardio-respiratory based physical activity for 30 minutes a day, five days a week, or alternatively perform vigorously intense cardio-respiratory based exercise 20 minutes a day, 3 days a week in addition to performing eight to 10 strength-training exercises, with eight to 12 repetitions of each exercise twice a week [2]. When prescribing exercise for mental health consumers such as those with PTSD, meeting the ACSM guidelines should be the ultimate aim. However ensuring engagement with the program regardless of how minimal it may be is likely to be rudimentary to its success, and can allow for progressions to be made as the participants become more confident and adherent to the program.

PTSD affects an estimated 5% of Australians, with hyperarousal, re-experiencing and avoidance the main symptom clusters [6]. Depression, anxiety, drug and alcohol addiction and sleep disturbance are common psychiatric comorbidities [6]. Treatment modalities include medications, cognitive behavioral therapy, psychodynamic psychotherapy, eye movement desensitization and reprocessing (EMDR) and group psychotherapy. Evidence-based treatment for PTSD is still quite limited and there is no definitive evidence to guide pharmacological prescription. The International Consensus Group on Depression and Anxiety recommends selective serotonin reuptake inhibitors (SSRIs) and exposure therapy [6]. The 2007 Australian Guidelines for the Treatment of Adults with Acute Stress Disorder and Post Traumatic Stress Disorder state that exercise may be helpful in managing symptoms and as part of self-care more generally [7], but the clinical guidelines note that no studies have examined the effectiveness of exercise as an adjunct to other PTSD treatments.

At the time of writing, no randomised controlled trial (RCTs) had been conducted investigating the effects of an individualized and structured exercise program on patients with a DSM-IV diagnosis of PTSD [7,8]. A 2010 Cochrane Collaboration review titled 'Sports and games for post-traumatic stress disorder found that no RCTs had been conducted to assess the effect of sports or game based interventions on symptoms of PTSD [8]. Although the review identified five studies, none met the inclusion criteria as they were not randomised controlled trials [9], participants were not diagnosed with PTSD or a psychological based intervention was tested such as play-therapy [10,11].

Some evidence of the potential benefit of exercise on PTSD symptoms comes from a 2008 study by Diaz and Motta [12]. They conducted a non-randomised study involving twelve female adolescents diagnosed with PTSD. Their results showed that 91% of participants showed a significant reduction in PTSD symptoms on the Childhood PTSD Symptom Scale, following participation in a walking program [12]. The study had a number of limitations including the use of a low intensity exercise protocol which did not include progressive overload training, and failed to incorporate any of the ACSM guidelines regarding exercise prescription. Given the potential impact on both the PTSD symptoms and physical co-morbidities, there is a compelling need for an evidence-based approach to prescribing exercise for people diagnosed with PTSD.

The current RCT will investigate the effects of structured, progressive exercise on PTSD symptoms, depression and anxiety, mobility and strength, body composition, physical activity levels, sleep patterns and medication usage among people with a DSM-IV diagnosis of PTSD.

## Methods/design

### Design

**Figure 1:**
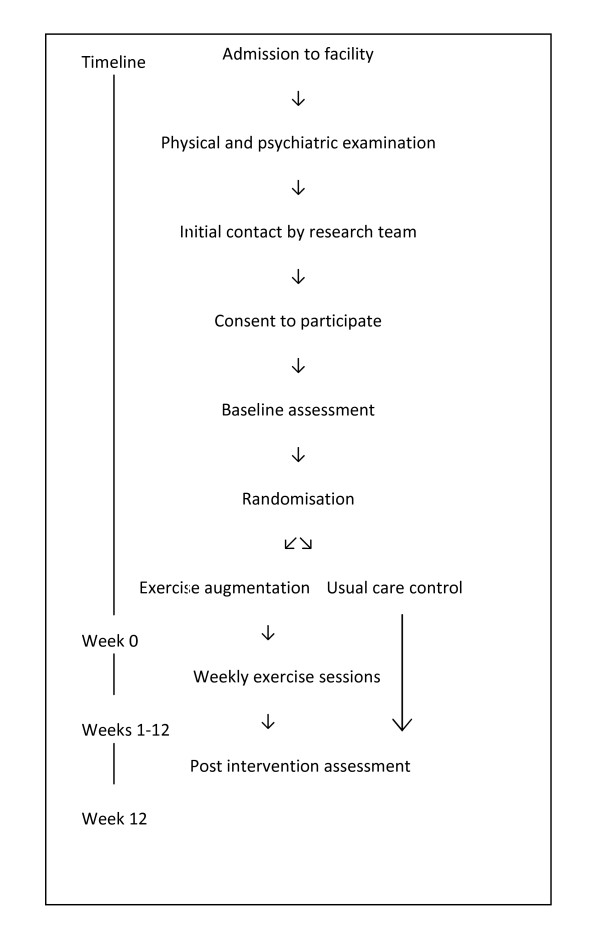
**Flow of participants through the trial**.

An assessor-blinded RCT will be conducted. A total of 40 participants in each group (n = 80) will be recruited. The study will have 80% power to detect as significant at the 5% level a 5 point between group difference on the PCL-C (SD = 9.4) [13] allowing 15% dropouts. The study protocol has been designed and will be reported, with reference to the CONSORT Statement [14]. Figure 1 gives an overview of the study design.

### Participants

Participants will be consenting people aged over 18 years with a DSM-IV diagnosis of primary PTSD. The study sample will consist of patients from St John of God Healthcare's Richmond Hospital, located in Sydney, Australia. To be eligible for study inclusion, participants must be considered medically fit to participate in an exercise program by the consulting medical officer. All participants must be cognitively able to provide consent.

People will be ineligible to participate in the trial if they are medically unfit to participate, are pregnant or planning pregnancy within the next year and if they are diagnosed with complex PTSD with trauma occurring in childhood only.

### Ethical considerations

Full ethical approval for this study has been obtained from St John of God Healthcares Ethical Committee (REF: 412). Written informed consent from all participants will be obtained prior to the baseline assessment. The research team will be advised by nursing and/or medical staff as to whether potential participants are cognitively able to provide consent.

### Randomisation

Upon admission to the hospital as either an in-patient or out-patient, a routine physical assessment will be conducted by a consulting medical officer to assess trial eligibility based on physical limitations. This initial assessment will also be used to confirm that the potential participant meets the DSM-IV diagnosis of PTSD. Eligible subjects will then be invited to participate in the trial by the research team. Posters, flyers and other marketing material will be distributed around the facility to promote awareness of the trial. Outpatients eligible to participate will be informed of the study through hospital staff at various times throughout their contact with the hospital.

Once consent has been obtained, the baseline assessment will be conducted and participants will then be randomised to either the exercise intervention or control group. Allocation to groups will be undertaken by a staff member not involved in recruitment (to ensure allocation concealment) using a block randomisation sequence generated using random numbers in Excel and including randomly varying block sizes. Participants and intervention staff are unable to be blinded to group allocation, but health and exercise professionals assessing all outcome measures will be blinded to group allocation.

### Intervention

The exercise intervention will comprise a minimum of three exercise sessions per week, with one session completed at the hospital under supervision of an exercise physiologist and the other two completed without supervision. All required exercise equipment will be supplied to participants for the duration of the study. The exercise protocol consists of a series of progressive compound exercises using exercise bands and body weight for resistance. Participants will perform 3 sets of 10 repetitions of each exercise. A warm-up set that is one intensity level below the 'overload sets' will also be included. A rest period of between 10 and 30 seconds will be allowed between sets. Once exercise technique is considered competent by the exercise physiologist or research nurse, participants will be advised to perform all exercises in a circuit like manner, adding a cardio-respiratory component to the exercise sessions. Exercises are outlined in Table 1.

**Table 1:** REAP Exercise Chart showing exercises and progression of intensity for each exercise

Exercise	Level 1	Level 2	Level 3	Level 4	Level 5	Level 6	Level 7
**^1^Chest press**	EB* 'light'	EB 'medium'	EB 'heavy'	EB 'extra heavy'	Incline push-ups	Knee push ups	Push ups

**^1^Row**	EB 'light'	EB 'medium'	EB 'heavy'	EB 'extra heavy'			

**^1^Squats**	Seated leg press (single leg)	Sit-to-stand	squats	EB 'light'	EB 'medium'	EB 'heavy'	EB 'extra heavy'

**^1^Core/mid section**	Wall hold	4-point kneeling	4-point kneeling with resistance	Prone hold			

**^2^Lat/overhead pull-down**	EB 'light'	EB 'medium'	EB 'heavy'	EB 'extra heavy'			

**^2^Upright row**	EB 'light'	EB 'medium'	EB 'heavy'	EB 'extra heavy'			

**^3^Bicep curl**	EB 'light'	EB 'medium'	EB 'heavy'	EB 'extra heavy'			

**^3^Triceps extension**	EB 'light'	EB 'medium'	EB 'heavy'	EB 'extra heavy'	Chair dips		

**^3^Leg extensions**	Body weight (single leg)	EB 'light'	EB 'medium'	EB 'heavy'	EB 'extra heavy'		

*EB exercise band

^1 ^Indicates 'Key Exercise'

^2 ^Indicates 'Secondary Exercise'

^3 ^Indicates 'Additional Exercise'

Each exercise session with the exercise physiologist will last for approximately 30 minutes. Sessions will be predominantly one-on-one, however during the later stages of the intervention it is expected that up to three participants could be present in a single exercise session.

During the initial exercise session, results from the physical assessment, International Physical Activity Questionnaire (IPAQ) [15] and the Borg Rating of Perceived Exertion Scale (RPE) [16] will be used to assign the initial program intensity from the Randomised Exercise Augmentation for PTSD (REAP) exercise table. It is expected that most of the cohort will be sedentary at the time of recruitment so exercises will be introduced in a staged manner in order to maximise adherence and technique acquisition. The REAP exercises have been segmented into 'Key', 'Secondary' and 'Additional' exercises indicating the order in which they will be introduced into the program. Individual limitations such as arthritic conditions, skill and coordination will also be considered when prescribing the exercises. For example if a participant is able to perform ten repetitions of the Level 1 chest press with an RPE below 12/20 (indicating light exertion), then the Level 2 progression will be attempted. Discretion will be used when applying the RPE scale. The weekly exercise sessions with the exercise physiologist/research nurse will be used to address any issues that participants may have with exercise technique, exercise progressions and adherence.

In addition to the resistance exercise program, a walking program will be included. Participants randomised to the intervention group will be provided with an Omron HJ109 pedometer in order to quantify daily step count. Participants will be asked to record their daily step count on the exercise diaries provided and the results of which will be used to assist with goal setting and motivation. Participants will be encouraged to aim for an ultimate daily target of 10,000 steps per day [17] which can be broken down into a series of short and incidental walks, in order to maximise feasibility and adherence.

Participants in the exercise group will also receive usual treatment for PTSD involving a combination of psychotherapy, pharmaceutical interventions and group therapy facilitated by psychologists. Voluntary programs including yoga, art therapy and use of the hospital gymnasium will also be available.

### Motivational Tools

Defined behaviour change techniques (BCT's) such as barrier identification, general encouragement, setting of graded tasks, instruction, specific goal setting, self-monitoring and feedback will be incorporated into the intervention program to enhance engagement and uptake of the intervention [18]. Exercise diaries and motivational interviewing will be a major component of the BCT's used. As recommended by the National Institute for Health and Clinical Excellence (NICE) 2007 guidelines on behaviour change at population, community and individual levels [19], the exercise physiologist and research nurse will use a combination of strategies to increase physical activity participation. Structured strategies to overcome common barriers to exercise will also be used. These strategies can be found in Table 2 [20]. Exercise diaries in the form of a printed spread-sheet will be provided and participants will be encouraged to record the date, time, number of sets and repetitions performed, and amount of walking done per day (measured with a pedometer). The diaries will also be used to guide progression of the intensity of individual exercise programs.

**Table 2:** Methods of improving behaviour change and overcoming barriers to exercise participation [19,20]

Ensure participants have an understanding of the short, medium and longer-term consequences of their health-related behaviours, for themselves and others
Assist participants to plan their behaviour changes in terms of easy steps over time

Recognise how social contexts and relationships may affect behaviour, and identify and plan for situations that might undermine changes being made

Plan explicit 'if-then' coping strategies to prevent relapse

Assist participants to make a personal commitment to adopt health-enhancing behaviours by setting (and recording) goals to undertake clearly defined behaviours, in particular contexts, over a specified time

Share their behaviour change goals with others

Encourage short bouts of exercise for participants with a 'lack of time'

Encourage group exercise sessions for participants with low motivation

Encourage moderate level activities if physical limitations are present

### Control Group

The control group will receive usual care, which will involve a combination of psychotherapy, pharmaceutical interventions and group therapy facilitated by psychologists. Access to these treatments by both the control and intervention groups will be recorded through use of the hospital database and patient files. Voluntary programs including yoga, art therapy and use of the hospital gymnasium will also be available. Access to the hospital exercise program is determined by the consulting medical physicians and the exercise physiologist. The control group will be asked to limit their participation in any exercise program that they do not usually undertake, however access to the usual hospital exercise program will not be restricted for the purpose of this research. The hospital exercise program involves two, one hour sessions per week in which patients have access to the gymnasium and an exercise physiologist. Equipment available includes treadmills, exercise bands and a limited number of machine weights. Self-initiated use of the hospital exercise program by in-patients with PTSD is usually limited, and it is not expected that this would have significant ramifications for the research. Participants randomised to the control group who wish to exercise will be permitted under the usual policy of the hospital, and participation will be monitored and recorded.

### Outcomes

#### Data Collection

Data will be collected from clinician interviews, self-report questionnaires, exercise, medication use and sleep diaries, and physical assessments. Health or exercise professionals collecting data will be blinded to group allocation. Physical assessments will be carried out at baseline, and following the 12 week intervention. All tests will be conducted in an examination room at the hospital. Assessments will take approximately 30 minutes to administer. All assessments will be carried out by an assessor blinded to group allocation.

### Outcome measures

#### Post Traumatic Stress Disorder symptoms

The primary aim of this study is to examine the effects of individualised, structured exercise on PTSD symptoms. This will be measured using the PTSD Checklist-Civilian [21], a self-report questionnaire comprising 17 items relating to the main symptoms of PTSD. Participants are asked to indicate how much they have been bothered by a particular symptom over the past month using a 5-point (1-5) scale.

#### Depression and Anxiety

The Depression Anxiety and Stress Scale (DASS) [22] will be used to assess the effects of the program on depressive and anxiety symptoms. The DASS is a 42-item self report instrument that measures the related negative emotional states of depression, anxiety and tension/stress.

#### Mobility, Fitness and Strength

Assessments of mobility will include tests of standing balance [23] (tandem, semi-tandem and single-leg stance time), the ability to rise from a chair, and the 6-minute walk test [24]. Upper limb strength will be assessed using a hand grip dynamometer with a single assessment to be carried out on each upper limb [25]. Knee extension strength will be assessed with a spring gauge attached to the participant's leg using a webbing strap with a Velcro fastener. The participant will extend their knee pulling against the strap with maximal force for 2-3 seconds with each leg to be tested 3 times and the best score recorded [26].

#### Body Composition

Measurements will include resting heart rate, blood pressure, weight, height, body mass index, body fat percentage (to be obtained using a Tanita bio-impedance scale), waist circumference (defined as the point midway between the iliac crest and costal margin) and hip circumference (defined as the widest part of the gluteal region).

#### Physical Activity Levels

Physical activity participation will be measured the short version of the IPAQ [15]. The IPAQ (short form) includes 7 questions relating to the amount of time spent per week engaging in vigorous and moderate physical activity, walking and sedentary activities.

#### Sleep Patterns

Sleep habits and patterns including amount of sleep hours per night and barriers to sleep will be monitored through the Pittsburgh Sleep Quality Index [27] and the Pittsburgh Sleep Quality Index Addendum for PTSD [28]. Both instruments are self-report questionnaires that assess sleep quality in the past month.

#### Medication Usage

Pro re nata (PRN) medication usage specifically sleeping and as required psychotropic medication will be measured using a weekly self-report diary, and patient notes whilst admitted as an inpatient.

### Statistical analysis

Regression models will be used to assess the effect of group allocation on the primary and secondary outcome measures after adjusting for baseline values. An intention-to-treat approach will be used for primary analyses. A secondary per-protocol analysis will be conducted excluding people who do not comply with the intervention, defined as performing less than 30% of the recommended exercise sessions. A further secondary analysis will assess whether there is an interaction between baseline physical activity levels and group allocation. Analyses will be conducted using the SPSS and Stata software packages.

## Discussion

The REAP study has been designed to fill a gap in the current scientific literature regarding the role of exercise augmentation for the treatment of PTSD. The study design will evaluate the dual role that exercise may play in both improving mental health outcomes, and improving overall cardio-metabolic risk and physical capacity.

The exercise protocol being trialed has been designed over a 12 month period through ongoing clinical exercise prescription and supervision with PTSD patients at St John of God Healthcare's Richmond hospital. The authors (program designers) have backgrounds in medicine, nursing, exercise science, epidemiology and physiotherapy. The protocol evolved from the need for a constant balance between assisting patients to engage in an exercise program, whilst making it achievable and affordable and able to be self-managed. Exercise bands have been selected as they provide an affordable, portable and safe alternative to traditional weights to progress exercise intensity. The assessments of physical abilities included in this trial were selected to be feasible for the research clinicians to conduct in addition to a psychiatric and physical examination within a constrained time period.

This study will determine the role of exercise augmentation for the treatment of primary PTSD. The results of this study will determine if an exercise program can be successfully implemented among people with PTSD. It will also provide a structured exercise protocol that can be replicated in other psychiatric facilities. This study may have implications for the perceived importance of clinical exercise within mainstream psychiatric facilities, and has the potential to lead to greater investment in the physical wellbeing of people with mental health conditions.

## Competing interests

The authors declare that they have no competing interests.

## Authors' contributions

SR, DN and TL conceived the idea and obtained funding for the study. All authors contributed to the design and development of the trial protocol. SR, CS and AT drafted the manuscript. All authors critically reviewed the manuscript and approved the final manuscript.

## Pre-publication history

The pre-publication history for this paper can be accessed here:

http://www.biomedcentral.com/1471-244X/11/115/prepub
